# T regulatory cells and attenuated bleomycin-induced fibrosis in lungs of CCR7^-/- ^mice

**DOI:** 10.1186/1755-1536-3-18

**Published:** 2010-09-03

**Authors:** Glenda Trujillo, Adam J Hartigan, Cory M Hogaboam

**Affiliations:** 1 Department of Pathology, University of Michigan, Ann Arbor, MI 48109-2200, USA

## Abstract

**Background:**

C-C chemokine receptor (CCR)7 is a regulator of dendritic cell and T cell migration, and its role in tissue wound healing has been investigated in various disease models. We have previously demonstrated that CCR7 and its ligand, chemokine (C-C motif) ligand (CCL)21, modulates wound repair in pulmonary fibrosis (PF) but the mechanism of this is unknown. The objective of this study was to investigate whether the absence of CCR7 protects against bleomycin (BLM)-induced PF. CCR7^-/- ^mice failed to mount a fibrotic pulmonary response as assessed by histologic collagen staining and quantification by hydroxyproline. We hypothesized that the prominent characteristics of CCR7^-/- ^mice, including elevated levels of cytokine and chemokine mediators and the presence of bronchus-associated lymphoid tissue (BALT) might be relevant to the protective phenotype.

**Results:**

Pulmonary fibrosis was induced in CCR7^+/+ ^and CCR7^-/- ^mice via a single intratracheal injection of BLM. We found that the lung cytokine/chemokine milieu associated with the absence of CCR7 correlated with an increase in BALT, and might be attributable to regulatory T cell (Treg) homeostasis and trafficking within the lungs and lymph nodes. In response to BLM challenge, CCR7^-/- ^mice exhibited an early, steady increase in lung CD4^+ ^T cells and increased CD4^+ ^CD25^+ ^FoxP3^+ ^Tregs in the lungs 21 days after challenge. These findings are consistent with increased lung expression of interleukin-2 and indoleamine 2,3-dioxygenase in CCR7^-/- ^mice, which promote Treg expansion.

**Conclusions:**

Our study demonstrates that the protective phenotype associated with BLM-treated CCR7^-/- ^mice correlates with the presence of BALT and the anchoring of Tregs in the lungs of CCR7^-/- ^mice. These data provide novel evidence to support the further investigation of CCR7-mediated Treg trafficking in the modulation of BLM-induced PF.

## Introduction

Idiopathic pulmonary fibrosis (IPF) is an irreversible, fatal lung disease, marked by progressive deterioration of lung function due to an uncontrolled repair process [1]. The pathogenesis of IPF is not completely understood, which is reflected in the lack of successful treatments and good diagnostic markers. Although it is generally accepted that IPF comprises an inflammatory and regenerative phase that results in excessive collagen deposition distorting normal lung architecture and function, the importance of inflammation in fibrosis has been challenged [2]. The majority of investigations aimed at dissecting the pathogenesis of fibrosis have been conducted in animal models, which have strongly suggested that the dysregulated healing process is sustained by chronic inflammation [3]. The inflammatory cells recruited to the lung and the factors they secrete dictate the nature, duration and severity of the inflammatory response. Chemokines and chemokine receptors are the primary regulators of the inflammatory cells that are (1) recruited to sites of injury (2) elicit wound-healing responses from the tissue resident cells.

C-C chemokine receptor (CCR)7 is a chemokine receptor that is expressed strongly on mature dendritic cells (DCs), naive and memory T cells, fibrocytes and structural cells comprising the fibroblastic foci in the lungs of patients with IPF [4-6]. The CCR7 ligands, chemokine (C-C motif) ligand (CCL)19 and 21, are constitutively expressed in the secondary lymphoid organs and lymphatic vessels, and are central to the homing and trafficking of T cells. CCR7 expression allows these cells to home to the lymph nodes (LNs), where they expand in response to antigen stimulation and suppress effector cell responses. The T cells traditionally associated with attenuating immune cell activation are the T regulatory cells (Tregs), but their role in fibrotic responses is not clear. The importance of CCR7 in proper Treg mobilization has been demonstrated by studies showing that Tregs lacking CCR7 accumulate in the lung and fail to traffic to the LNs [7,8].

We have previously demonstrated that CCR7 and CCL21 are key regulators in the pathogenesis of PF [6,9,10]. In the study we report here, we targeted CCR7 in a bleomycin (BLM) model of PF, which has been useful in dissecting the roles of the chemokine/cytokine network in both the inflammatory and fibrogenic phases of lung fibrosis. We conducted our investigation in the CCR7^-/- ^mouse, which possesses bronchus-associated lymphoid tissue (BALT) and demonstrates impaired homing of Tregs [7,8]. BALT is often described as a tertiary lymphoid tissue rich in B and T cells. Although its exact function is not clearly understood, several murine studies have implicated BALT in facilitating primary immune responses via local priming of lymphocytes and in serving as a reservoir for viruses during their latency period [11,12]. The importance of lymphocytes in the pathogenesis IPF remains controversial; however, lymphocyte infiltration in areas of active fibroblastic proliferation in the IPF have been identified and shown to exhibit an elevated CD4/CD8 T cell ratio that correlates positively with survival of these patients [13-15].

In this study, we hypothesized that CCR7^-/- ^mice would be protected from the deleterious pulmonary effects of BLM. We assessed the histologic effects of BLM in the lungs, and investigated the chemokine/cytokine milieu in the lungs of wild type (WT) and CCR7^-/- ^mice. Our data suggest that the increased levels of inflammatory cell infiltrates in the BALT of CCR7^-/- ^mice may protect against fibrosis. Further, these infiltrates correlated with the impaired trafficking of Tregs between the lungs and LNs during the inflammatory and fibrogenic phases of BLM-induced PF. We found Tregs accumulating in the lungs of both CCR7 gene-deleted mice. Our data support the further investigation of the underappreciated role of T cells and inflammation in the pathogenesis and maintenance of PF.

## Results

### BLM-induced PF is diminished in CCR7^-/- ^mice compared with WT

**Figure 1:**
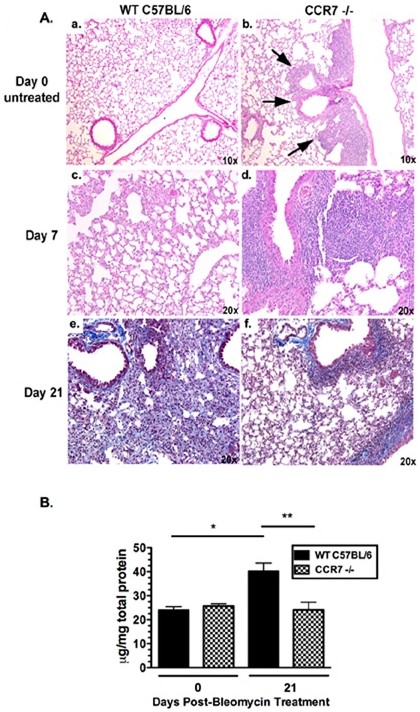
**CCR7**^-/- ^**mice exhibit decreased collagen deposition in response to bleomycin**. **(A) **Representative histologic sections of mouse lungs. Mouse lung from **(a) wild type (**WT) and **(b) **C-C chemokine receptor (CCR)7^-/- ^mouse 21 days after bleomycin treatment. Masson-trichrome stain. **(B) **Hydroxyproline levels in whole lung homogenates from untreated or bleomycin-challenged mice. Data are mean ± SEM from four to five mice at each time point, and are representative of three separate experiments. **P < 0.01.

In the present study, we investigated whether the absence of CCR7 affects the development of PF in a murine model. WT (CCR7^+/+^) and CCR7^-/- ^mice received a single intratracheal injection of BLM on day 0, and we compared the degree of fibrosis in the lungs 21 days later. Similar to previous reports [16], histologic analysis indicated severe lung fibrosis in BLM-treated WT mice, as evidenced by complete distortion of normal lung architecture, interstitial thickening, fibroblastic proliferation and abundant Masson-trichrome staining for collagen, (Figure 1A panel a). This effect was not seen in the lungs of BLM-treated CCR7^-/- ^mice, which had significantly less histologic fibrosis (Figure 1A panel b). The lungs of BLM-treated CCR7^-/- ^mice, analyzed at day 21, exhibited decreased staining for collagen and retention of the integrity of the alveolar space (Figure 1Ae, 1Af). These histologic differences were confirmed by the quantification of hydroxyproline, an index of total lung collagen and fibrosis: on day 21 after BLM injection, the lungs of CCR7^-/- ^mice had significantly less collagen-associated hydroxyproline compared with those of WT mice (Figure 1B).

### Expansion of BALT occurs in the lungs of BLM-treated CCR7^-/- ^mice during the early inflammatory phase

CCR7^-/- ^mice have been previously described as possessing highly organized BALT and severely reduced numbers of Tregs in the lung-draining (bronchial) (br)LNs [8]. Figure 1A confirms the presence of BALT localized to the main bronchi and arteries (black arrows), as previously described in CCR7-deficient mice (Figure 1Ab) which was absent in untreated WT mice (Figure 1Aa) [8]. We next analyzed lung histopathology in BLM-treated mice to determine whether histologic changes occur in the BALT within the first 7 days after the BLM insult. Hematoxylin and eosin staining of lung tissue obtained from mice at day 7 after BLM injection indicated that WT lungs demonstrate a typical robust inflammation in the alveolar space (Figure 1Ac), which appeared augmented in the lungs of CCR7^-/- ^mice because of the apparent expansion of the lymphoid tissue surrounding the bronchi (Figure 1Ad). Interestingly, BALT in the lungs of CCR7^-/- ^mice did not appear to be in an expanded state by day 21 after BLM injection (Figure 1Af), suggesting that the pulmonary inflammation in CCR7^-/- ^mice is tightly regulated despite the constitutive presence of immune cell aggregates.

**Figure 2:**
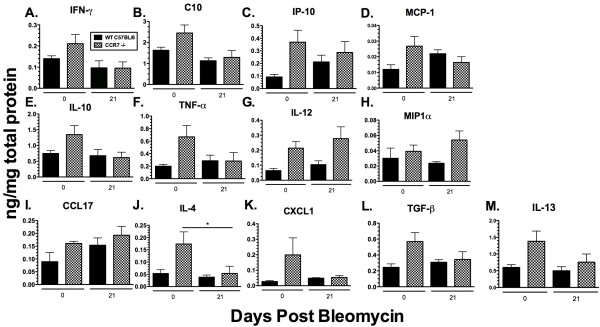
**Global Increase in inflammatory mediators observed in lungs of untreated CCR7**^-/- ^**mice**. ELISA analysis of (**A**) interferon (IFN)γ, (**B**) C10, (**C**) IFN-inducible protein (IP)-10, (**D**) monocyte chemotactic protein (MCP)-1, (**E**) interleukin (IL)-10, (**F**) tumor necrosis factor-α, (**G**) IL-12, (**H**) monocyte chemotactic protein (MIP)1-α, (**I**) chemokine (C-C motif) ligand (CCL)17, (**J**) IL-4, (**K**) CXC chemokine ligand (CXCL)1, (**L**) transforming growth factor-β and (**M**) IL-13 in whole lung homogenates from untreated wild type (WT) and C-C chemokine receptor (CCR)7^-/- ^mice and in WT and CCR7^-/- ^mice at day 21 after bleomycin challenge. Data are mean ± SEM from four to five mice at each time point. *P < 0.1).

To test whether the histologic differences observed between WT and CCR7^-/- ^mice (Figure 1) correlate with differences in the production of inflammatory mediators, we next quantified both Th1 and Th2 pro- and anti-inflammatory cytokines in whole lung homogenates by ELISA 21 days after BLM injection. Interestingly, the data demonstrated that even before BLM challenge, untreated (day 0) CCR7^-/- ^mouse lung homogenates exhibited an overall striking increase in Th1- and Th2-associated cytokines and chemokines compared with untreated WT lungs (Figure 2). Although the differences did not reach significance, the overall trend was consistent for all the cytokines and chemokines tested. These data correlate with the presence of BALT in untreated (day 0) CCR7^-/- ^mice (Figure 1Ab). By day 21 after BLM injection, protein levels in CCR7^-/- ^lungs were comparable or lower than those measured in the WT lungs, with the exception of interleukin (IL)-12 and macrophage inflammatory protein (MIP)1α, which are slightly increased in CCR7^-/- ^lung homogenates compared with WT lungs and have been implicated in BLM-induced PF. No significant differences were detected in the levels of inflammatory mediators between WT and CCR7^-/- ^mouse lungs on day 21 after BLM injection, suggesting that CCR7^-/- ^mice do have increased production of pro- or anti-inflammatory mediators. Taken together, these data indicate that the protective phenotype exhibited by CCR7^-/- ^mice is not related to an increased inflammatory response after BLM injection, but is possibly due to an increase in the lymphocyte population in the lung that is localized within the BALT.

### Intrapulmonary fibrocyte recruitment is unchanged in BLM-treated CCR7^-/- ^mice

**Figure 3:**
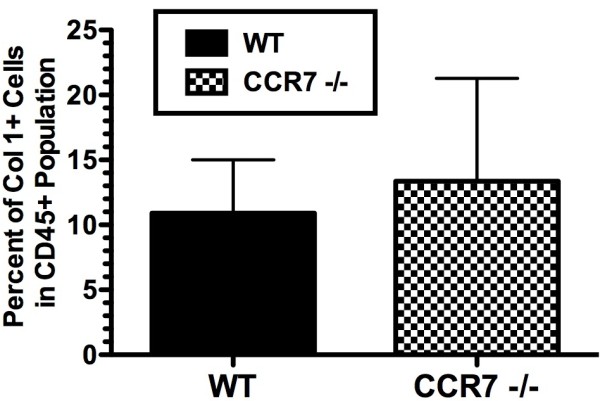
**Fibrocyte recruitment to the lungs in bleomycin-treated WT and C-C chemokine receptor (CCR)7**^-/- ^**mice**. Flow cytometry analysis of fibrocytes in whole lungs of wild type (WT) and CCR7^-/- ^mice 7 days after bleomycin challenge. Cells were immunostained with phycoerythrin (PE)-conjugated anti-mouse CD45 and biotin-conjugated anti-mouse collagen 1 and allophycocyanin-labeled strepavidin. Data shown are the percentages of collagen 1+ cells of total CD45+ population from five mice in each group.

The recruitment of fibrocytes to the lung during inflammation has been documented as an important factor in the development of PF, and is mediated, in part, by CCR7 and its ligand, CCL21 [17,18]. We next determined whether fibrocyte recruitment to the lung was altered in CCR7^-/- ^mice compared with WT mice during the peak inflammatory phase of BLM-induced PF. In line with the methods of previous studies, we quantified the percentage of Col 1^+ ^CD45^+ ^cells in whole lung homogenates from WT and CCR7^-/- ^mice 7 days after BLM challenge, to determine the number of fibrocytes present in the lung during the peak inflammatory phase of BLM-induced injury. BLM increased the number of intrapulmonary fibrocytes to a similar extent in WT and CCR7^-/- ^mice (Figure 3), suggesting that the attenuated fibrotic response in BLM-treated CCR7^-/- ^mice was independent of fibrocyte recruitment to the lung and possibly mediated by other cell types.

### CD4^+^CD25^hi+^FoxP3^+ ^cells are present in lungs of CCR7^-/- ^mice but absent in WT mice during the fibrotic stage of BLM-induced injury

**Figure 4:**
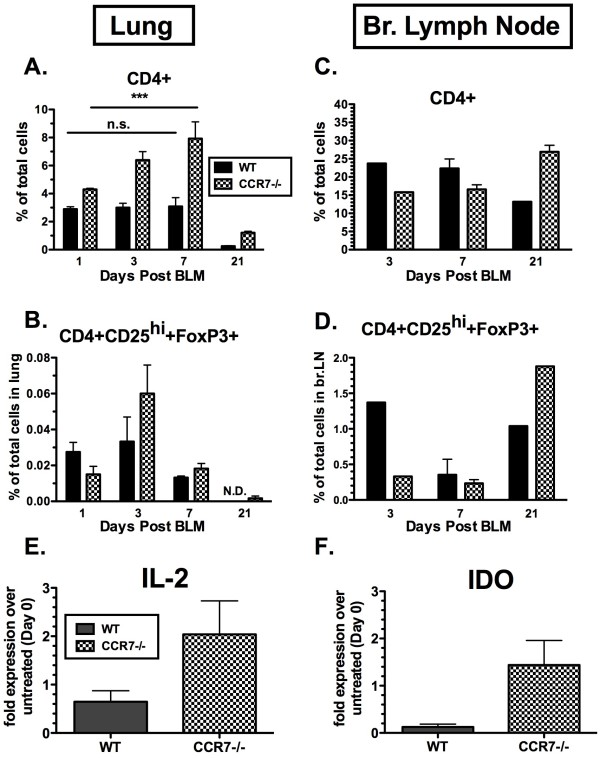
**Absence of CCR7 is associated with pulmonary localization of CD4+, CD4+CD25+FoxP3+ and CD8+ T cells**. (**A, B**) Lung cells and (**C, D**) bronchial lymph node cells from wild type (WT) and C-C chemokine receptor (CCR)7^-/- ^mice 1,3,7 and 21 days after bleomycin challenge were stained with (**A, C**) phycoerythrin (PE)-cyanin (Cy)7-conjugated anti-CD4 or (**B, D**)PE-conjugated anti-CD25 and PE-Cy5-conjugated anti-forkhead box (FOX)P3. CD4+CD25+FOXP3 T cells are expressed as percentage of total cells. n = three to mice/group/experiment. Samples were individualized except in those from the bronchial lymph nodes (brLNs) on days 3 and 21, in which cells from three to five mice were pooled. These data are representative of two separate experiments. TaqMan PCR analysis of whole lung samples for (**E) **interleukin (IL)-2 and (**F**) indoleamine 2,3 dioxygenase (IDO) transcript expression 21 days after bleomycin challenge. Transcript expression was compared with that before the intratracheal (IT) bleomycin challenge (that is, day 0). All data are shown as the expression fold difference from three to five mice at each time point after i.t. bleomycin challenge. SEM from four to five mice at each time point. ***P < 0.001. ND = not detected; NS = not significant.

Distinct T cell areas have been described in BALT, which have been identified as having an important role in DC interactions and cytokine production [19-21]. Because we observed an expansion of BALT in the lungs of CCR7^-/- ^mice, we next determined the T lymphocyte populations in the lung and lung draining LNs after BLM challenge. Analysis of total CD4^+ ^cells indicated a steady increase in their percentage in the lungs of CCR7^-/- ^mice during the peak inflammatory response after BLM injection (Figure 4A). This trend was not observed in the lungs of WT mice at the same time points, (that is, days 1, 3 and 7 after BLM injection) (Figure 4A). At day 21 after BLM injection, a decrease in CD4^+ ^cells was observed in the lungs of both WT and CCR7^-/- ^mice, but the percentage of CD4^+ ^cells in CCR7^-/- ^lungs still exceeded that in WT lungs (Figure 4A). Previous studies have demonstrated that mice deficient in CCR7 exhibit a significant reduction in the numbers of Tregs in the lung-draining LNs and impaired homing of Tregs into secondary lymphoid organs [8]. Therefore, we next investigated whether differences in Treg numbers between WT and CCR7^-/- ^lungs at day 21 correlated with the histologic differences observed (Figure 1). One day after BLM injection, we detected fewer Tregs (CD4^+^CD25^hi+^FoxP3^+^) in the lungs of CCR7^-/- ^mice compared with WT; however, throughout days 3 and 7 after BLM injection, the change in the percentage of CD4^+ ^CD25^hi+ ^FoxP3^+ ^cells was similar in both WT and CCR7^-/- ^mice (Figure 4B). By contrast, 21 days after BLM injection, Tregs were not detected in the lungs of WT mice, but were maintained at low levels in CCR7^-/- ^mice (Figure 4B). Localization of FoxP3-expressing Tregs in the BALT of CCR7^-/- ^mice was confirmed by immunohistochemistry (data not shown). Furthermore, we determined that on day 21, gene transcript expression of the Treg-promoting mediators, IL-2 (Figure 4E) and indoleamine 2,3 dioxygenase (IDO) (Figure 4F), were also increased in the lungs of CCR7^-/- ^mice compared with WT.

CCR7 expression by T cells mediates their trafficking between the lung and draining LN, where expansion and maintenance of the T cell memory pool occurs. In a murine model of allergic airway inflammation, CCR7 was shown to control the exit of T cells from peripheral tissues and entry into afferent lymphatics [22]. To determine whether the lack of CCR7 localized T cells to the lungs of CCR7^-/- ^mice and prevented the exit into the draining LNs, we examined the accumulation of T cells in the lung draining brLNs in WT and CCR7^-/- ^mice during the inflammatory phase (days 3 and 7) and the fibrotic phase (day 21) after BLM injection. The percentages of CD4^+ ^cells on days 3 and 7 after BLM injection were less in the brLNs from CCR7^-/- ^mice compared with WT, although by day 21 the percentage of CD4^+ ^cells in the brLN of CCR7^-/- ^mice exceeded that of WT (Figure 4C). These data were not surprising because CCR7 has been identified as a required homing receptor for T cell exit from the lung and entry to the draining lymphatics [22]. CCR7 has also been shown to be important for the migration of Tregs into the peripheral LNs, where they proliferate and exert effector cell functions [7]. As mentioned above, we found a small percentage of CD4^+^CD25^hi+^FoxP3^+ ^Tregs in the lungs of BLM-treated CCR7^-/- ^mice on day 21, which were undetectable in the lungs of BLM-treated WT mice, indicating that the absence of CCR7 contributes to the retention of Tregs in the inflamed lung. Interestingly, we also detected Tregs in the brLNs of BLM-treated CCR7^-/- ^mice (Figure 4D), suggesting that an alternative mechanism functions to localize Tregs in the LN in the absence of CCR7.

## Discussion

The purpose of our study was to determine whether the absence of CCR7 attenuated PF in mice that were challenged intratracheally with BLM. We report that CCR7^-/- ^mice have decreased sensitivity to BLM-induced injury in the lung because they fail to exhibit the severe pulmonary remodeling typically associated with BLM-induced PF. In this study we present a complete profile of cytokine expression in WT and CCR7^-/- ^mice at baseline and after BLM challenge, and an examination of fibrocytes and immune cells into the lung. Although we acknowledge that significant differences were not quantifiable under our experimental conditions, we are confident about the striking, reproducible trends we have observed.

Although CCR7 is strongly expressed in various bone marrow-derived cells, most notably DCs, we focused our study on T cell trafficking in BLM-induced PF because an intrinsic characteristic of CCR7^-/- ^mice is the presence of BALT in their lungs. BALT is a tertiary lymphoid structure that functions as a local reservoir for priming T lymphocytes and is involved in the impaired homing of Tregs in CCR7^-/- ^mice [7,8,12]. We observed that the BALT in CCR7^-/- ^mice was altered during the course of BLM-induced injury, and may represent the dynamics of Treg trafficking during BLM-induced pulmonary remodeling.

Treg cells represent a conservative 1-10% of total CD4^+ ^T cells, yet their immunosuppressive functions are indispensable for immune homeostasis [23]. Our study demonstrates that Treg-promoting genes (IL-2 and IDO) are upregulated in the lungs of BLM-treated CCR7^-/- ^mice, which suggests that the lung environment in CCR7^-/- ^mice is favorable to the maintenance of a Treg cell population. Furthermore, we found that CD4^+^CD25^hi+^FoxP3^+ ^Treg cells are retained in the lungs of BLM-treated CCR7^-/- ^mice, which is consistent with an ameliorated remodeling response to BLM-induced injury in the lung. Preliminary studies using CCR7^-/- ^bone-marrow chimeras confirmed the localization of CCR7^-/- ^Tregs to the lung, which we consider is due to a CCR7 deficiency in hematopoietic cells and not to altered functions of the structural cells within the lungs of CCR7^-/- ^mice (GT, AJH, CMH, unpublished data). These ongoing studies also indicated an appreciable increase in the total number of CD4^+ ^cells in the lungs of chimeric mice (CCR7^-/-^→WT) during the fibrotic phase at 21 days after BLM injection (GT, AJH, CMH, unpublished data), which is consistent with that in the intact CCR7 knockout mice. The Treg population in the lungs of chimeric mice (CCR7^-/-^→WT) was also increased compared with WT controls (WT→WT), an effect previously observed in BLM-treated CCR7^-/- ^mice, and Tregs were present in the LNs of CCR7^-/- ^chimeric mice 21 days after BLM injection challenge. Taken together, our results present novel evidence for an underappreciated role for CCR7 in the context of Treg trafficking during the pathogenesis of PF.

We observed an increase of BALT in the lungs of CCR7^-/- ^during BLM-induced PF and we believe that this promotes protection from BLM-induced PF. Tertiary lymphoid structures in the lung have been implicated in providing sustained protective immunity to viruses and chronic asthma, and in lung cancers, tumor-induced BALT has been associated with increased survival [11,21,24]. Similarly, lymphoid aggregates resembling lymphoid follicles have been identified in fibrosing diseases, including IPF [25-27], although its exact function and origin is not known. We found that the BALT is a dynamic structure within the lungs of CCR7^-/- ^mice, and its function during BLM-induced PF may be related to the retention of Tregs in the lung.

A recurring observation in our study that warrants further investigation is CCR7-independent trafficking during PF. Firstly, CCR7 has been widely shown to function as a potent stimulus for chemotaxis of bone marrow-derived Col 1-positive cells (fibrocytes) [18]. Our study did not indicate significant diminution of fibrocytes in BLM-treated CCR7^-/- ^mice despite their protection from fibrosis. These data were not entirely surprising, because recruitment of fibrocytes to murine lungs has been shown to be mediated by other receptors including chemokine CXC motif receptor (CXCR)4, CCR2 and CCR5 [28-30]. Although we did not analyze the expression of these receptors in our system, it is plausible that they may function as a compensatory mechanism to recruit fibrocytes to the lungs of CCR7^-/- ^mice. Moreover, we cannot discount the possibility that fibrocyte proliferation in the lung may also account for the comparable levels of fibrocytes observed in WT and CCR7^-/- ^mice.

Second, contrary to our expected results and those previously reported by Kocks *et al*., we consistently detected relatively high levels of CD4^+^CD25^hi+^FoxP3^+ ^Tregs in the brLN of CCR7^-/- ^mice on days 3 and 7, and in fact a higher percentage of CD4^+ ^and CD4^+^CD25^hi+^FoxP3^+ ^Tregs in CCR7^-/- ^mice (compared with WT) 21 days after BLM injection. These results could be indicative of compensatory, CCR7-independent mechanisms that regulate the trafficking of Tregs from the lungs to the brLNs. Such mechanisms have been proposed in the context of the trafficking of CD4^+ ^T cell to the brLNs of CCR7-deficient mice during influenza infection, the presence of CD11b^+^CD11c^+ ^DCs in the mediastinal LNs of CCR7-deficient mice during the late phase of *Mycobacterium tuberculosis *infection, and the homing of central memory CD8^+ ^T cells to peripheral LNs in *plt/plt *(CCL19 and CCL21-Ser-gene deleted) mice [31-33]. In addition, Zou *et al. *previously demonstrated that human CD4^+^CD25^+ ^Tregs traffic and are retained in the bone marrow through CXCR4/CXCL12 (CXC chemokine ligand 12) [34]. Therefore, we cannot exclude the possibility that our detection of CD4^+^CD25^hi+^FoxP3^+ ^Tregs in brLNs of BLM-treated CCR7^-/- ^mice in this study is related to these CCR7-independent pathways.

During the preparation of this manuscript, a study by Kotsianidis *et al. *was published, which reported a dramatic decrease in the number and function of Tregs in both bronchoalveolar lavage and peripheral blood from patients with IPF [35]. The data presented in our current study contribute to the current understanding of the role of Tregs during PF. T cell expansion has clinical potential for treating a variety of diseases such as autoimmunity and chronic asthma, and can be manipulated to promote graft-specific tolerance during organ transplantation. Therapeutic injections of IL-2 monoclonal antibodies have been shown to expand the Treg population *in vivo *without inducing proliferation of CD8^+ ^and NK cells [36]. Moreover, therapeutic transfer of regulatory T cells prevents development of allergic airway remodeling [37]. Our study provides a rationale for targeting CCR7 in IPF and for the further investigation of therapies to favor the expansion and retention of the Treg population in the IPF lung.

## Conclusions

The data presented in this study indicate a potential protective role for BALT and retention of Tregs in the lung during BLM-induced PF. We found that CCR7 is linked to Treg cell trafficking and the amelioration of BLM-induced PF. These data provide a rationale for further studies investigating CCR7 and the regulation of Treg trafficking as potential therapeutic targets against IPF.

## Methods

The Animal Use Committee at the University of Michigan (Ann Arbor, MI, USA) approved all protocols and experiments described in this report.

### Mice

Specific-pathogen-free male C57BL/6 (WT; CCR7^+/+^) mice (6 to 8 weeks of age) were purchased from Taconic (Germantown, NY, USA). CCR7^-/- ^mice were generated as previously described[38], and were bred and housed under specific pathogen-free conditions.

### BLM model of pulmonary inflammation and fibrosis

WT (CCR7^+/+^), CCR7^-/- ^mice, or bone-marrow chimeras were given 0.05 U of sterile BLM sulfate (Blenoxane, Bristol-Meyers Pharmaceuticals, Evansville, IN, USA) dissolved in phosphate-buffered saline (approximately 1.7 U/kg body weight) via an intratracheal injection. Groups of WT (CCR7^+/+^), CCR7^-/- ^mice (n = 5-10 per timepoint) and bone-marrow chimeras were monitored for survival. Mice in other groups were killed and their lung tissues analyzed at days 1, 3, 7 and 21 after BLM injection. Untreated mice (n = 5) did not receive BLM, and this time point was designated as day 0.

### Hydroxyproline assay

Left lobe samples from WT (CCR7^+/+^) and CCR7^-/- ^mice (n = 5 per group for each time point in each experiment) before (that is, day 0) and at day 21 after BLM challenge were analyzed for hydroxyproline using a previously described assay [16,39].

### Cytokine and chemokine ELISA analysis

Screening for the chemokines/cytokines C10, interferon (IFN)-γ, IFN-inducible protein-10, monocyte chemotactic protein (MCP)-1, macrophage inflammatory protein (MIP)1α, IL-4, IL-10, IL-12, IL-13, CCL17, CXCL1, tumor necrosis factor-α and transforming growth factor-β was carried out on 50 μl samples of cell-free supernatants from whole lung homogenates using a standardized sandwich ELISA technique (R&D Systems, Minneapolis, MN, USA). Cytokine and chemokine levels in each sample were normalized to the protein present in cell-free preparation of each sample, as measured by the Bradford protein assay.

### Flow cytometry analysis

Lung cells were isolated after collagenase dispersion to obtain single-cell suspensions, as previously described [40], and single cell suspensions of lung-draining LNs were prepared by isolation of bronchial LNs, pushing cells through a 40 μm nylon mesh using a syringe. Cells were stained with the indicated antibodies after 5 min of preincubation with Fc receptor blocking reagent (Fc Block; BD Biosciences, San Jose, CA, USA) fixed overnight with 4% formalin and run on a Cytomics FC-500 (Beckman Coulter, Brea, CA, USA). Samples were analyzed using FlowJo software (Tree Star, Inc., Ashland, OR, USA).

### Real-time TaqMan PCR analysis

Total RNA was prepared from whole lung samples using TRIzol reagent according to the manufacturer's directions (Invitrogen, Carlsbad, CA, USA). A total of 1.0 μg from whole lung RNA was reverse transcribed into cDNA using Moloney murine leukemia virus reverse transcriptase as described previously (Invitrogen) [16]. TaqMan gene expression reagents were used to assay IL-2 and IDO (Applied Biosystems, Foster City, CA, USA). Whole lung gene expression in WT (CCR7^+/+^) and CCR7^-/- ^mice was expressed as a fold increase in transcript expression in BLM-challenged lung compared with unchallenged lung. The fold difference in mRNA expression between treatment groups was determined by software developed by Applied Biosystems.

### Statistical analysis

All results are expressed as mean ± SEM. The means between groups at different time points were compared by two-way ANOVA. Individual differences were further analyzed using the unpaired *t*-test with Welch correction or the Tukey-Kramer multiple comparisons test where indicated. Values of *P *< 0.1*, *P *< 0.01** and *P *< 0.001*** were considered significant.

## Competing interests

The authors declare that they have no competing interests.

## Authors' contributions

GT and AJH carried out the experiments and data analysis. GT, AJH and CMH designed the experiments and interpreted the data, and GT wrote the final manuscript. All authors read and approved the final manuscript.
